# Web‐Based Application of Simplified Machine Learning for Detecting Reduced LVEF From 12‐Lead ECG

**DOI:** 10.1002/joa3.70296

**Published:** 2026-02-20

**Authors:** Hiroshi Kawakami, Yohei Doi, Kazumichi Yamamoto, Yan Luo, Makoto Saito, Katsuji Inoue, Osamu Yamaguchi

**Affiliations:** ^1^ Department of Cardiology, Pulmonology, Nephrology and Hypertension Ehime University Graduate School of Medicine Toon Japan; ^2^ Department of Nephrology Osaka University Graduate School of Medicine Suita Japan; ^3^ Department of Health Promotion and Human Behavior Kyoto University Graduate School of Medicine, School of Public Health Kyoto Japan; ^4^ Medical Education Center Kyoto University Graduate School of Medicine Kyoto Japan; ^5^ Department of Cardiology Kitaishikai Hospital Ozu Japan

**Keywords:** diagnostic method, electrocardiography, left ventricular ejection fraction

## Abstract

**Background:**

Deep learning (DL) models have shown high accuracy in detecting reduced left ventricular ejection fraction (LVEF) from electrocardiograms (ECGs). However, their complexity limits clinical use. To address this, we aimed to develop and validate simplified machine learning (ML) models using numerical parameters from 12‐lead ECGs to detect LVEF < 40% and to implement them in a user‐friendly web application.

**Methods:**

We retrospectively analyzed ECG and transthoracic echocardiography data from 21 471 patients across two institutions. The dataset was divided into a development cohort (non‐atrial fibrillation [non‐AF]: *n* = 12 922; AF: *n* = 1281) and an external validation cohort (non‐AF: *n* = 6284; AF: *n* = 984). Four machine learning algorithms—random forest (RF), extreme gradient boosting (XGBoost), support vector machine, and generalized additive models with LASSO—were evaluated for predicting LVEF as a continuous variable and binary outcome (< 40%).

**Results:**

For continuous LVEF prediction, RF achieved *R* ^2^ values of 0.68 (non‐AF) and 0.74 (AF) in internal validation but performed poorly in external validation. Other models showed *R* ^2^ values below 0.40 in internal validation. For binary classification, all models achieved area under the curve (AUC) values > 0.90 in the non‐AF group during internal validation. RF and XGBoost showed strong performance in the AF group (AUC > 0.90 internally) and adequate accuracy externally (AUCs of 0.80–0.81 in AF and 0.90 in non‐AF).

**Conclusions:**

We developed a simple web‐based tool for preliminary screening of reduced LVEF using 12‐lead ECG parameters.

## Graphical abstract

A simple web‐based application enables rapid screening for reduced left ventricular ejection fraction (LVEF < 40%) using automatically measured 12‐lead ECG parameters. This machine learning–based tool supports noninvasive preliminary assessment of systolic dysfunction in both sinus rhythm and atrial fibrillation.

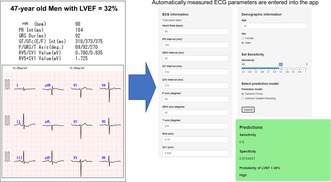

**Abbreviations**
AI: artificial intelligence
AUC: area under the curve
DL: deep learning
GAMLASSO: generalized additive models with LASSO
ML: machine learning
RF: random forest
RMSE: root mean square error
SVM: support vector machine
XGBoost: extreme gradient boosting

## 1 Introduction

The left ventricular ejection fraction (LVEF), a pivotal indicator associated with comprehensive systolic left ventricular function, is the most established metric for cardiac function assessment. Representing the percentage of blood ejected during the systole, a normal cardiac LVEF ranges from approximately 50–70%, and values below 50% are indicative of left ventricular systolic dysfunction [1, 2]. In contemporary heart failure management, LVEF serves as a cornerstone for disease classification and plays a decisive role in treatment strategy determination [3, 4]. Furthermore, LVEF is the most important indicator of future complications in patients with cardiovascular disease [3, 4]. In addition to cardiovascular medicine, LVEF has extensive utility in risk assessment for non‐cardiac surgeries and pre‐chemotherapy evaluations involving cardiotoxic agents [5, 6].

In the assessment of LVEF, measurement using transthoracic echocardiography (TTE) has been widely accepted as the most established and useful method because of its noninvasive nature and high versatility. However, an accurate evaluation requires the expertise of a proficient cardiologist or a trained physiological testing technician [1, 2, 7, 8, 9]. In addition, echocardiography requires approximately 15–30 min per examination and imposes substantial human and time‐related burdens, presenting challenges for the expeditious determination of LVEF in routine clinical settings [5, 10].

Recently, several studies have attempted to predict the LVEF using 12‐lead electrocardiograms (ECGs), which are widely accessible and simplified diagnostic tool [11, 12, 13, 14, 15, 16, 17, 18, 19, 20, 21]. These studies indicated that the application of artificial intelligence (AI) leveraging machine learning (ML) techniques to 12‐lead ECG data can aid in detecting patients with a reduced LVEF [12, 13, 14, 15, 16, 17, 18, 19]. Although some of these studies have included external validation, the performance is frequently reported in settings where the training and validation cohorts are relatively similar in patient characteristics, ECG acquisition systems, and echocardiographic measurement protocols. In addition, most existing models require large numbers of ECG‐derived features or raw waveform data, necessitating specialized data formats, advanced signal processing, and substantial computational resources. While such approaches can optimize predictive accuracy, they may limit reproducibility and hinder deployment in everyday clinical environments, particularly in facilities without advanced ECG analysis infrastructure.

To address these issues, we aimed to develop a simple and interpretable machine learning (ML) model for detecting LVEF ≤ 40% using only 10 ECG parameters automatically measured by commercially available ECG machines, combined with patient age and sex. Our goal was to create a user‐friendly tool that could be readily applied in clinical settings. We also evaluated its performance across different institutions and patient populations. Separate models were developed for atrial fibrillation (AF) and non‐AF groups to account for potential rhythm‐specific differences.

## 2 Methods

In the present study, we developed a model to predict the LVEF using 12‐lead ECG data. We followed the Transparent Reporting of a multivariable prediction model for Individual Prognosis or Diagnosis Statement for the development and validation of the prediction model [22, 23]. The study protocol was approved by the Institutional Review Board of Ehime University Graduate School of Medicine (approval no.: 2312006). The authors confirm that patient consent was not applicable to this study.

### 2.1 Study Population

**FIGURE 1:**
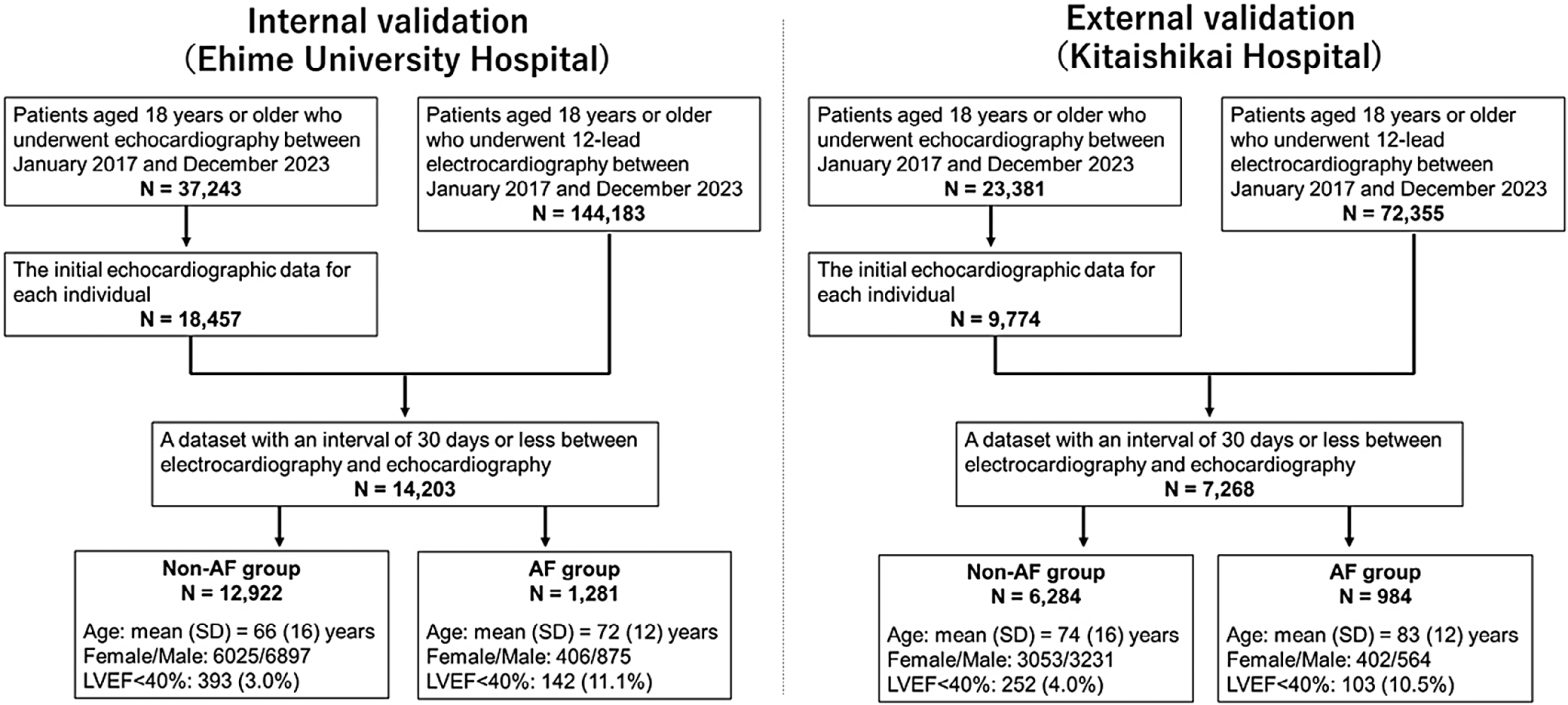
Study population and participant recruitment flowchart.

A flowchart of participant recruitment is shown in Figure 1. We retrospectively screened all consecutive patients who underwent both ECG and TTE at Ehime University Hospital between March 27, 2017, and June 30, 2023. We then selected patients who were at least 18 years old at the time of TTE and had their LVEF evaluated using the modified Simpson method. Subsequently, the inaugural cardiac echocardiographic records for each participant were isolated and integrated with the corresponding electrocardiographic data using a unique identifier. Further refinement involved the extraction of instances in which the temporal gap between electrocardiographic and echocardiographic examinations did not exceed 30 days. Finally, within each dataset, we identified the recording pair with the shortest time interval between the ECG and TTE assessments. The external validation set was derived using data in Kitaishikai Hospital between March 27, 2017, and June 30, 2023. Patients were selected using the same methodology described above. To explicitly evaluate inter‐center transportability, we predefined an institution‐wise split: one institution was reserved as the external test cohort, while model training and internal assessment were performed using data from the other institution.

### 2.2 12‐Lead ECG and TTE

All 12‐lead ECGs were acquired at a sampling rate of 500 Hz in a 10‐s period using ECG devices manufactured by Nihon Kohden (Tokyo, Japan). The model numbers for the Nihon Kohden ECGs used in this study were as follows: ECG‐1350, ECG‐1450, ECG‐1550, ECG‐2450, and ECG‐2550. All TTE examinations were performed by experienced cardiologists or sonographers using high‐end cardiovascular ultrasound in accordance with the recommendations of the American Society of Echocardiography [1, 24].

### 2.3 Study Outcome

The primary objective of this study was to develop predictive models for the LVEF, both as a continuous and binary outcome, with a defined cutoff value of 40%. The LVEF was evaluated by TTE using the modified Simpson method [1, 24].

### 2.4 Missing Data

The ECG parameters were automatically calculated by the devices, and no missing values were observed except for the P‐axis and P‐R intervals. Upon investigation, we found that the majority of patients with missing P‐axis and P‐R interval values had atrial fibrillation (AF). These deficiencies were considered informative, and multiple imputations were not performed. To establish the predictive model, the participants were divided into two groups: those with complete ECG parameters (non‐AF group) and those with deficiencies in the P‐axis and P‐R interval (AF group). To account for these differences, predictive models were constructed separately for each group. All ECG information used in this study was automatically generated by the device, including RV5 and SV1, without any manual measurement.

### 2.5 Exploratory Analysis of Correlations and Relationships

To further understand the dataset and inform model development, we conducted an exploratory analysis of parameter correlations and their relationships with the LVEF. Correlation analysis: Pairwise correlations among the included parameters were evaluated using Pearson's correlation coefficient. Scatter plots were generated to visualize the relationships between individual ECG parameters and LVEF, analyze the strength and direction of these associations, and identify potential predictors of LVEF. Association with the LVEF: To assess the relationship between individual parameters and LVEF, scatter plots were created and locally estimated scatterplot smoothing was performed. These analyses were used to evaluate the predictive potential of each parameter for continuous LVEF outcomes.

### 2.6 Model Development

The following 12 independent variables were considered: age, sex, and 10 parameters from the 12‐lead ECG data (heart rate, P‐R interval, QRS duration, corrected QT interval, corrected QT interval [QTc], P‐axis, QRS axis, T‐axis, RV5 voltage, and SV1 voltage). These ECG parameters are measurable automatically using standard 12‐lead ECG. For our modeling approach, we employed four distinct ML methodologies encompassing both regression and classification tasks, each embodying unique conceptual frameworks: a generalized additive model with LASSO (GAMLASSO), support vector machine (SVM) with a radial kernel, random forest (RF), and extreme gradient boosting (XGBoost). GAMLASSO extends the GAM framework by incorporating variable selection and regularization, effectively reducing overfitting and enhancing model interpretability. In the GAMLASSO methodology, parameter standardization is performed as a preprocessing step. The SVM is a powerful supervised learning algorithm known for its effectiveness in classification tasks. We chose the radial kernel owing to its ability to handle nonlinear relationships between the features and the target variables. Parameter standardization is applied as a preprocessing step for the SVM. RF and XGBoost represent ensemble tree‐based approaches: RF combines multiple decision trees through bagging to improve model stability and interpretability, whereas XGBoost performs sequential boosting with built‐in L1/L2 regularization and tree‐depth control to mitigate overfitting. These two methods are conceptually complementary, providing robustness through different ensemble mechanisms. Hyperparameter tuning for each algorithm was performed using grid search within a 10‐fold cross‐validation framework. To ensure internal robustness, the cross‐validation procedure was repeated with 100 bootstrap resamples. In this framework, the area under the ROC curve (AUC) was computed from the combined out‐of‐fold predictions, providing a single pooled estimate of discrimination. Fold‐specific AUCs were also examined to confirm internal consistency across validation folds. This approach ensures full use of the validation data while maintaining independence between training and testing folds.

In this study, the final model selection from each development stage was determined based on the results obtained from a grid search of the hyperparameters for each method.

### 2.7 Model Performance

To evaluate the predictive performance of the models for continuous LVEF, the *R* ^2^ statistic and root mean square error (RMSE) were used. For the binary LVEF classification (≥ 40% or < 40%), calibration was assessed using calibration plots. The discriminatory ability of the model was evaluated using the receiver operating characteristic curve analysis, with the area under the curve (AUC) as a key metric. As described in the “Missing Data” section, conditional model branching was applied based on the presence or absence of P‐axis and P‐R interval deficiencies in the dataset. To provide a consistent assessment across methods, internal discrimination and calibration were derived from the same resampling framework described above, ensuring methodological comparability among all models.

### 2.8 Model Validation

A bootstrap resampling procedure was performed for 100 iterations. Additionally, repeated 10‐fold cross‐validation was incorporated to evaluate the stability of model estimates and quantify internal optimism. For continuous LVEF outcomes, the optimism‐corrected *R* ^2^ was calculated, whereas for binary outcomes, the optimism in the AUC and calibration slope was quantified. External validation was performed using an independent dataset from Kitaishikai Hospital, assessing the *R* ^2^, RMSE, AUC, and calibration slope to evaluate the generalizability of the model (Figure 1). Accordingly, the trained models were directly applied to the held‐out institution for external validation. This external evaluation verified the robustness of model performance across institutions, with careful separation of development and validation datasets to prevent data leakage.

### 2.9 Sample Size Calculation

In general, a minimum of 10 and 20 participants are recommended for logistic and linear regression analyses, respectively, per independent variable. However, ML techniques may require a larger sample size, typically 10 times more events for each predictor variable than classical modeling techniques, such as logistic regression [25]. Moreover, more than 200 events per predictor parameter may prove to be inadequate, particularly for models with more than 12 parameters where sample sizes exceeding 120 and 240 are deemed necessary. In this study, we utilized a sample size of more than 11 000 participants, which corresponds to 1000 events per predictor, a figure we deemed acceptable for robust model development.

### 2.10 Statistical Software

We used R (version 4.2.1; R Foundation for Statistical Computing, Vienna, Austria) for all analyses. The final prediction model is presented on a website using the R Shiny application.

## 3 Results

### 3.1 Study Population

Figure 1 illustrates the participant recruitment and dataset construction processes. The final dataset was divided into two cohorts: [1] the model development and internal validation Cohort, consisting of 12 922 participants in the non‐AF group and 1281 participants in the AF group, derived from Ehime University Hospital, a large academic center, and (2) the external validation cohort, which included 6284 participants in the non‐AF group and 984 participants in the AF group, sourced from Kitaishikai Hospital, a regional community hospital. The patient selection criteria and methodological approaches were identical across both cohorts, and all datasets were handled independently in order to minimize the risk of overlap or inadvertent data leakage. Age, sex distribution, and proportion of participants with an LVEF of < 40% in each group are shown in Figure 1. The proportion of patients with an LVEF of < 40% was similar between the internal and external validation cohorts in both groups. However, participants in the external validation cohort were approximately 10 years older on average than those in the internal validation cohort.

### 3.2 Correlation Analysis Between the Parameters

**FIGURE 2:**
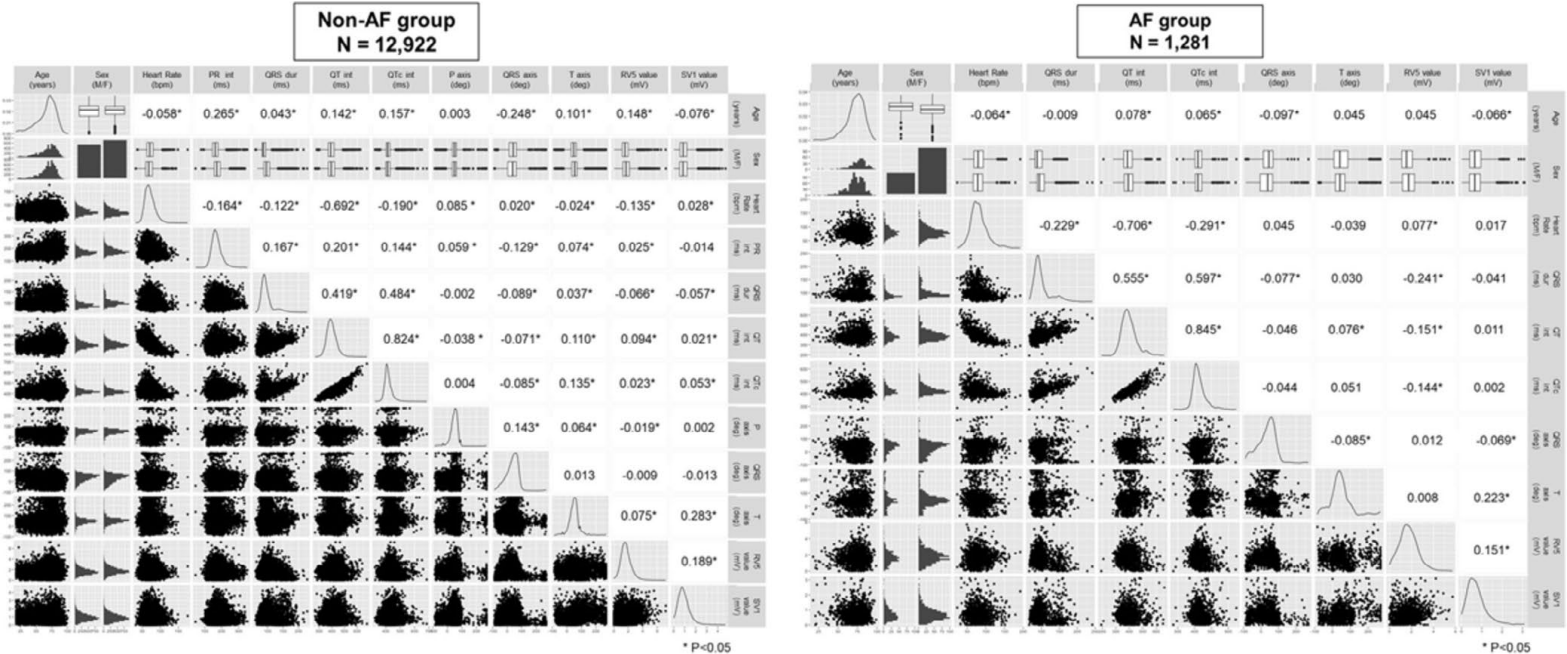
Correlation matrix of ECG parameters. Pearson correlation coefficients are used to display the relationships between various ECG parameters in the study population. ECG, electrocardiography.

Figure 2 presents the correlation matrix of the included parameters evaluated using Pearson correlation coefficients. Although numerous significant correlations were observed, parameters with a correlation coefficient exceeding 0.6 were limited. Strong correlations were primarily observed in inherently related parameters, such as the heart rate, QT interval, and QTc. In addition, the results differed between the non‐AF and AF groups, reflecting variations in the underlying parameter distributions.

### 3.3 Relationship Between Parameters and LVEF

Supplementary Figure 1 illustrates the relationships between individual parameters and LVEF analyzed using scatter plots and locally estimated scatterplot smoothing. The QRS duration demonstrated an inverse relationship with the LVEF, with wider QRS durations corresponding to a lower LVEF in both the non‐AF and AF groups. The other parameters showed limited and less discernible associations with the LVEF.

### 3.4 LVEF Prediction as a Continuous Variable

For continuous LVEF prediction, four machine learning models (GAMLASSO, SVM, RF, and XGBoost) were trained and validated (Table 1 and Supplementary Figure 2). The performance metrics included the *R* ^2^ and RMSE (Table 1). Among the four models, RF demonstrated a relatively better performance in the internal validation, with *R* ^2^ values reaching 0.68 and RMSE values of approximately 5.2 in the development dataset. However, its performance markedly decreased in the external validation, with *R* ^2^ values as low as 0.22 and RMSE exceeding 8.0. The other three models—GAMLASSO, SVM, and XGBoost—consistently showed very low *R* ^2^ values, typically below 0.40 in internal validation. In the external validation, the performances of these models were particularly poor, with all *R* ^2^ values falling below 0.30, indicating a severe lack of predictive capability across the datasets. High RMSE values were observed across all models, further emphasizing the difficulty in accurately predicting the LVEF as a continuous variable. These results indicate that none of the four models is practical for predicting the LVEF as a continuous variable in this study.

**TABLE 1:** Results of regression analyses with continuous variables as outcomes.

Non‐AF group	Internal validation (*N* = 12 922)		External validation (*N* = 6284)	
Machine learning methodologies	R‐Squared	RMSE	R‐Squared	RMSE
GAMLASSO	0.18	8.4	0.02	9.2
SVM	0.28	7.8	0.13	8.6
RF	0.68	5.2	0.22	8.2
XGBoost	0.40	7.2	0.22	8.2

AF group	Internal validation(*N* = 1281)		External validation (*N* = 984)	
Machine learning methodologies	R‐Squared	RMSE	R‐Squared	RMSE
GAMLASSO	0.18	11.4	< 0.01	11.2
SVM	0.22	11.1	0.04	11.0
RF	0.74	6.4	0.12	10.6
XGBoost	0.34	11.2	0.10	10.7

Abbreviations: AF, atrial fibrillation; GAMLASSO, generalized additive models with least absolute shrinkage and selection operator; RF, random forest; RMSE, root mean square error; SVM, support vector machine; XGBoost, extreme gradient boosting.

### 3.5 Classification of LVEF < 40%

**FIGURE 3:**
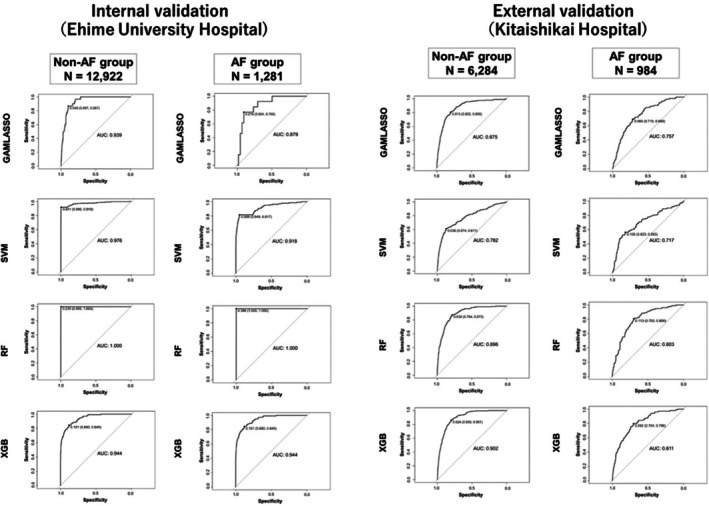
Performance of machine learning models for binary classification (LVEF < 40%). The figure presents the area under the curve (AUC) values of random forest (RF), extreme gradient boosting (XGBoost), support vector machine (SVM), and generalized additive models with LASSO (GAMLASSO) in internal and external validations. LVEF, left ventricular ejection fraction.

**FIGURE 4:**
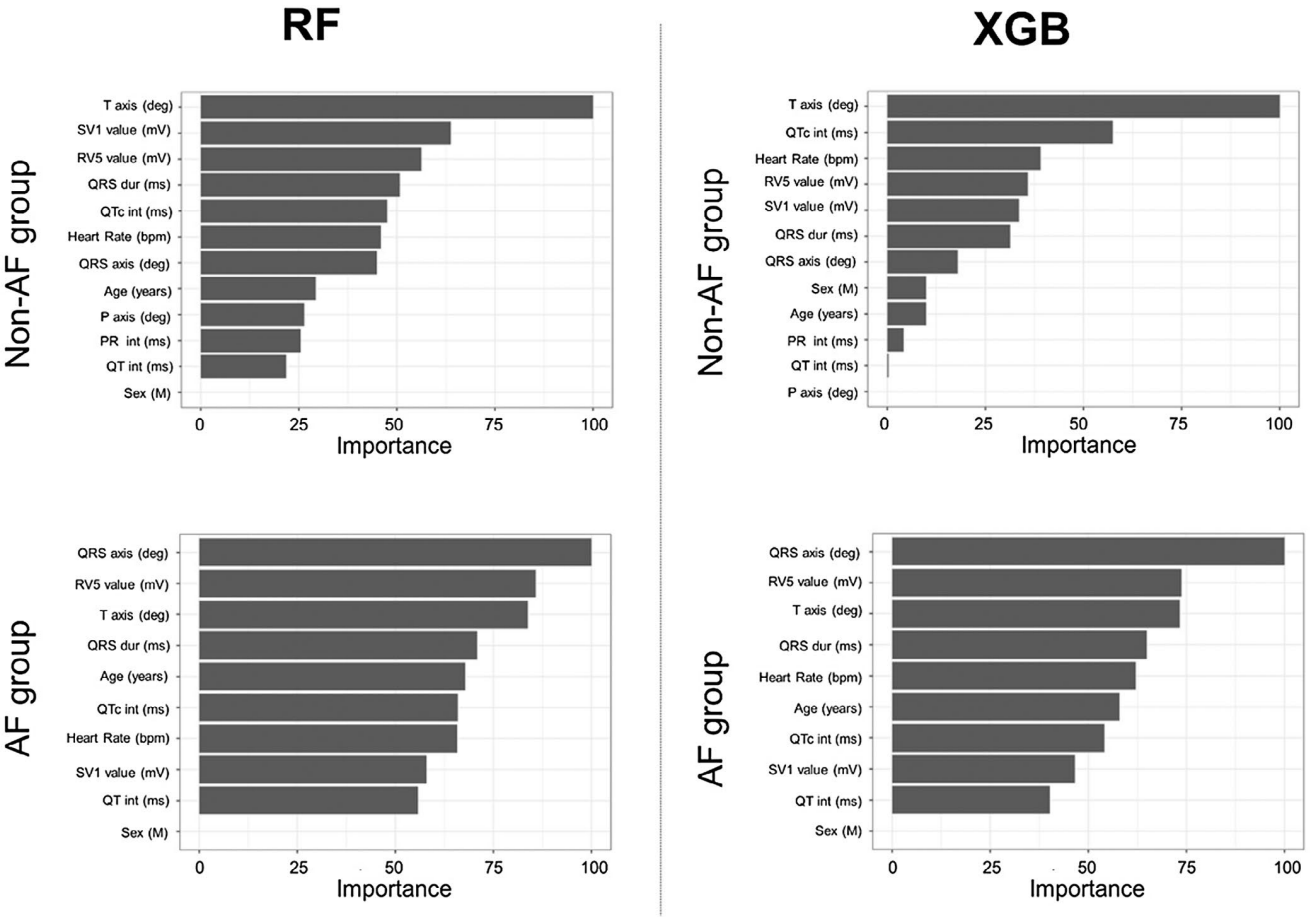
Feature importance of random forest and XGBoost models.

**FIGURE 5:**
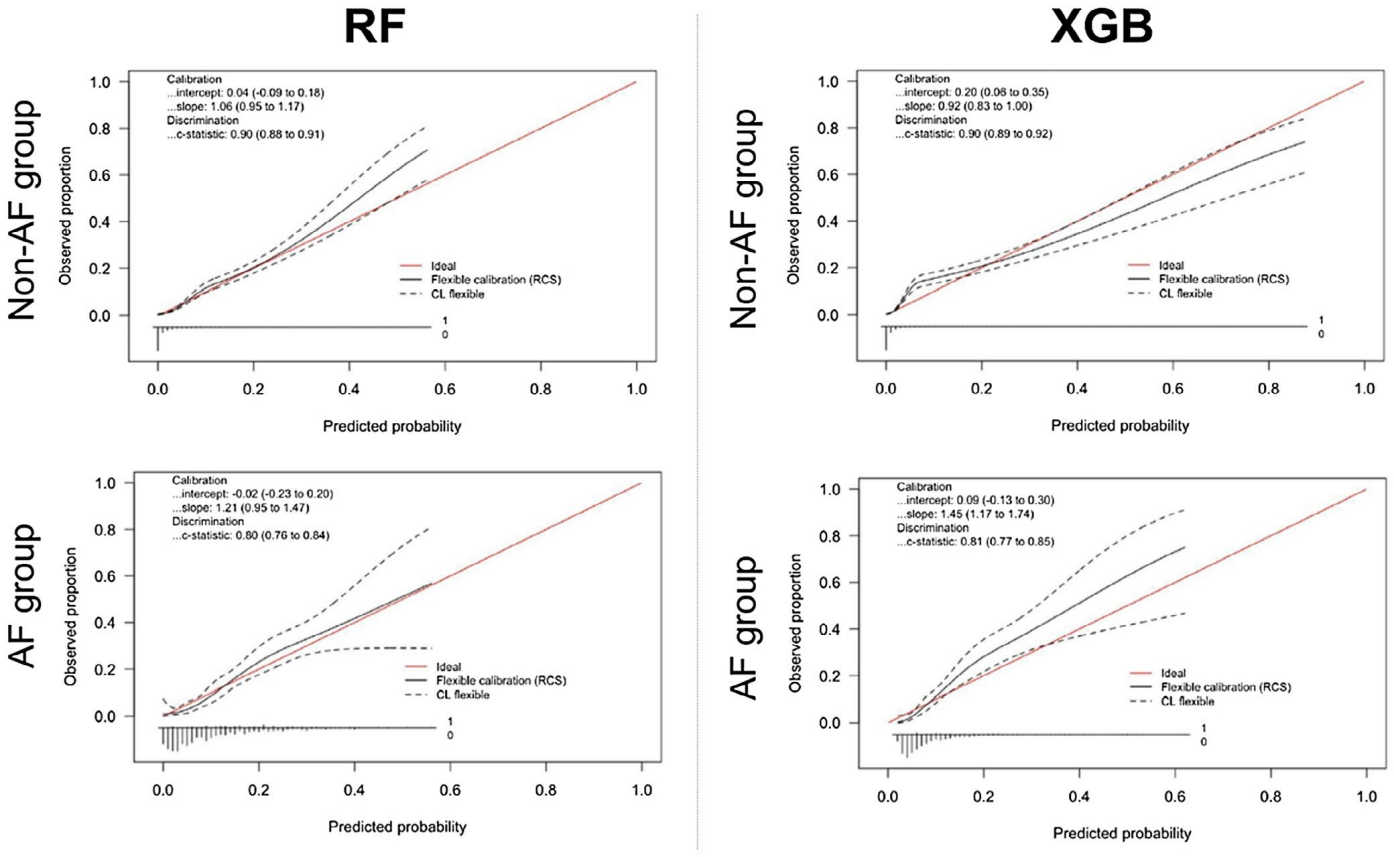
Calibration curves for binary classification models in the external validation. The alignment between predicted probabilities and observed outcomes is shown for RF and XGBoost models. RF, random forest; XGBoost, extreme gradient boosting.

The results of the binary classification models for predicting an LVEF of < 40% are summarized in Table 2 and Figure 3. Overall, the models demonstrated strong discriminatory performance in the internal validation, with consistently high AUC values. In the non‐AF group, all models achieved AUC values exceeding 0.90, ranging from 0.93–1.00. In the AF group, all models except GAMLASSO recorded AUC values above 0.90, indicating favorable predictive performance for RF, XGBoost, and SVM in this cohort. To further examine the AUC of 1.00 observed for the RF model, fold‐specific AUCs from 10‐fold cross‐validation were evaluated. The fold‐level AUCs ranged from 0.86 to 0.94 (mean ± SD = 0.91 ± 0.03; median [IQR] = 0.91 [0.90–0.92]), indicating that no single fold achieved complete discrimination and that the pooled AUC of 1.00 resulted from the aggregation of all out‐of‐fold predictions rather than reflecting true class separability (Supplementary Table S1). Given the substantial class imbalance and the strong physiological relationships between ECG‐derived features and reduced LVEF, this should be interpreted as a statistically plausible but extreme outcome of the pooled ROC computation rather than evidence of absolute classification performance. Univariate ROC analyses of each ECG feature showed only moderate predictive ability (AUC range: 0.38–0.77; Supplementary Table S2), supporting that the model's performance did not depend on any single variable but rather on the integrated contribution from multiple ECG‐derived parameters. In the external validation, a general decline in AUC values was observed across all models. However, RF and XGBoost demonstrated relatively favorable predictive performances compared with the other models. Specifically, RF achieved an AUC of 0.90 in the non‐AF group and 0.80 in the AF group, whereas XGBoost showed consistent results with AUCs of 0.90 in the non‐AF group and 0.81 in the AF group. These external results suggest that the near‐perfect AUC observed in internal validation was not fully explained by data leakage or procedural overfitting, and was also influenced by the characteristics of the underlying data structure and the ensemble learning process. These results highlight the comparative robustness of RF and XGBoost across independent institutional datasets, despite the overall decline in performance. Based on these findings, RF and XGBoost were selected as candidates for implementation in the application, and additional detailed validation was conducted. Figure 4 illustrates the importance of each factor in predicting an LVEF of < 40% for the candidate models, RF and XGBoost. The feature importance represents the relative contribution of individual predictors to the classification accuracy of the model. Calibrations of RF and XGBoost were evaluated using external validation data to assess their reliability in predicting an LVEF of < 40%. Figure 5 shows the calibration plots for both models. In the non‐AF group, RF achieved a calibration slope of 1.06, whereas XGBoost demonstrated a slope of 0.92, indicating good agreement between the predicted probabilities and observed outcomes. In the AF group, the calibration slopes were slightly higher, with RF and XGBoost showing slopes of 1.21 and 1.45, respectively. Although these values indicate slightly poorer calibration compared with that of the non‐AF group, they remain within a range that suggests practical applicability.

**TABLE 2:** Performance at LVEF (< 40%) classification.

Non‐AF group	Internal validation (*N* = 12 922)		External validation (*N* = 6284)	
Machine learning methodologies	AUC	Calibration slope	AUC	Calibration slope
GAMLASSO	0.93	1.14	0.88	0.93
SVM	0.98	3.47	0.78	1.00
RF	1.00	NA	0.90	1.06
XGBoost	0.94	2.65	0.90	0.92

AF group	Internal validation (*N* = 1281)		External validation (*N* = 984)	
Machine learning methodologies	AUC	Calibration slope	AUC	Calibration slope
GAMLASSO	0.88	1.30	0.76	0.88
SVM	0.92	3.07	0.72	1.08
RF	1.00	NA	0.80	1.21
XGBoost	0.94	2.65	0.81	1.45

Abbreviations: AF, atrial fibrillation; AUC, area under the curve; LVEF, left ventricular ejection fraction; GAMLASSO, generalized additive models with least absolute shrinkage and selection operator; RF, random forest; svm, support vector machine; xgboost, extreme gradient boosting.

### 3.6 Application Development

**FIGURE 6:**
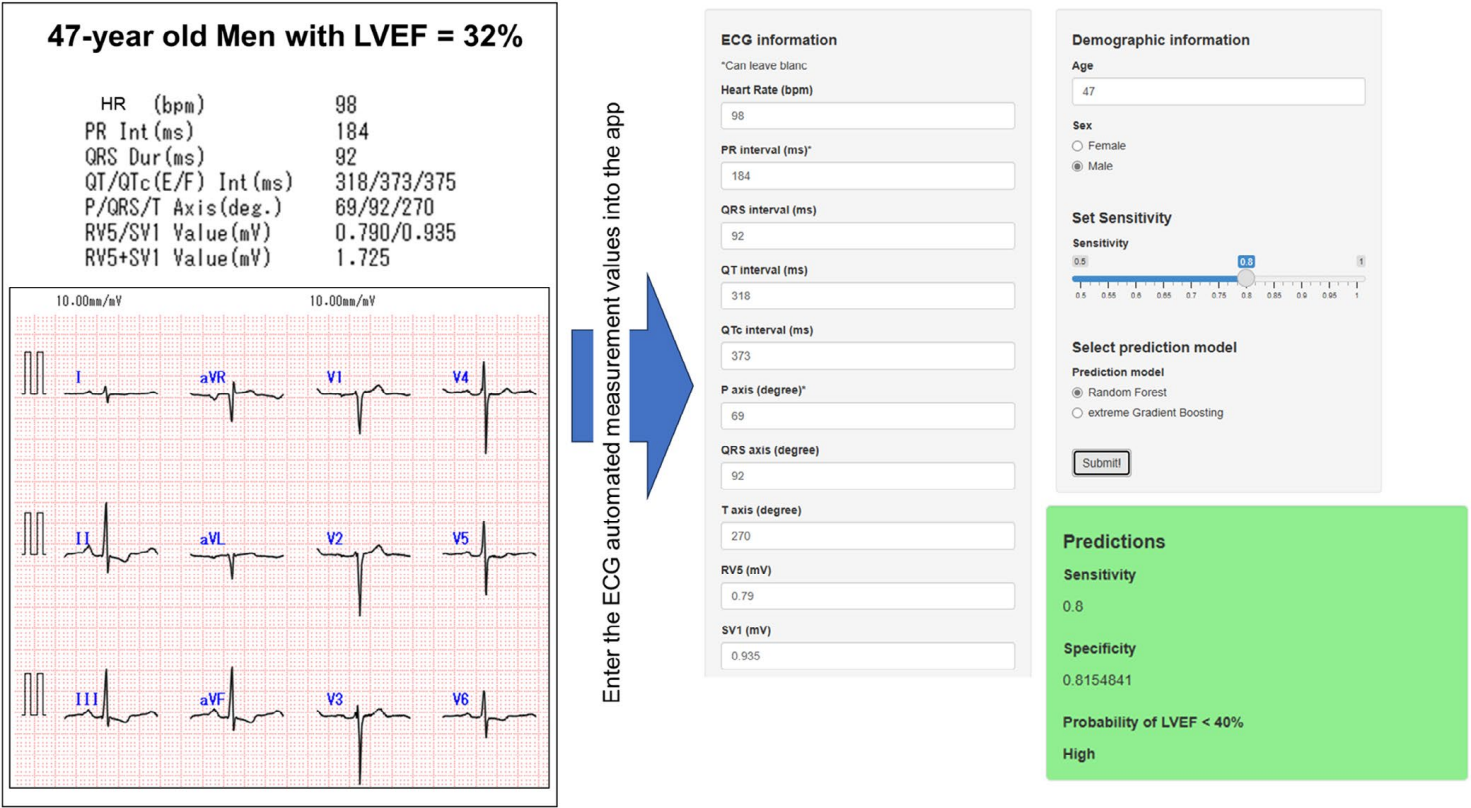
Example of the web‐based application interface. Age, sex, and 10 automatically derived ECG parameters are entered, and the predicted probability of LVEF < 40% is displayed. ECG, electrocardiography; LVEF, left ventricular ejection fraction.

We developed a web‐based application implementing the RF and XGBoost models to predict an LVEF < 40% (Figure 6; https://ehime‐university‐cardiology.shinyapps.io/ECG_HeartFailure_Predictor/). The application accepts age, sex, and 10 automatically derived ECG parameters, and allows users to choose between RF and XGBoost, which were selected based on their superior performance in external validation. The output provides the estimated probability of an LVEF < 40%, together with sensitivity and specificity, which can be adjusted to the desired threshold. As an illustration, Figure 6 presents an example case of a patient with reduced LVEF, showing how the application interface operates in practice.

## 4 Discussion

In this study, we developed and validated predictive models for LVEF using 12‐lead ECG parameters and classical ML algorithms. Continuous LVEF prediction remained difficult, with lower generalizability in the external validation. By contrast, classification for detecting LVEF < 40% showed robust performance, with AUC values consistently exceeding 0.90 in the internal validation. Prediction was feasible using only age, sex, and 10 automatically provided ECG parameters. The models also performed reasonably well in patients with atrial fibrillation, although the relative contribution of predictors appeared to differ from that in non‐AF patients. We also developed a web‐based application implementing these models. These findings should be interpreted in light of the vendor‐specific nature of the ECG data.

### 4.1 Use of AI for LVEF Prediction

Recently, AI has shown promise in leveraging 12‐lead ECG data to predict the LVEF [12, 13, 14, 15, 16, 17, 18, 19, 20]. Several studies have reported the diagnostic potential of AI‐enabled ECG (AI‐ECG) models and demonstrated their high predictive accuracy. A recent systematic review reported that AI‐ECG models achieved an AUC exceeding 0.90 in identifying reduced LVEF, suggesting good discrimination across different populations. Although the LVEF cutoff used to define reduced systolic function varies among studies, these findings underscore the growing applicability of AI‐based approaches in cardiovascular risk stratification [20]. Furthermore, previous studies have emphasized the broader implications of AI‐ECG models for risk stratification [12, 16, 17, 20]. Patients identified as high risk by AI‐ECG models often exhibit elevated long‐term risks for heart failure and cardiovascular events, even in cases of false positives [12, 16, 17, 20]. This suggests that AI‐ECG models not only facilitate the early detection of a reduced LVEF but also capture subclinical markers of future systolic dysfunction.

### 4.2 Differences in Regression and Classification Models

In this study, regression models for continuous LVEF prediction performed markedly worse than classification models for detecting LVEF < 40%. Several factors may explain this discrepancy. First, the relatively small number of patients with LVEF < 40% created a class imbalance that disproportionately impaired regression accuracy. Second, the relationship between ECG parameters and LVEF is not strictly linear, making continuous prediction prone to error, particularly around the cutoff of 40%, where small deviations can markedly worsen RMSE. By contrast, classification models are less affected by distribution skewness because they focus on discriminating values above or below a threshold. This likely explains their higher AUCs and greater robustness in our analysis. In particular, the RF model yielded an AUC of 1.00 during internal validation. Additional analyses showed that this value represented a statistically plausible but extreme outcome of pooled out‐of‐fold ROC computation under substantial class imbalance and strong physiological feature associations, rather than evidence of absolute discrimination. Importantly, individual fold‐level AUCs ranged from 0.86 to 0.94, indicating that no single fold achieved complete separation. Furthermore, although no evidence of data leakage or procedural overfitting was identified, the internal AUC = 1.00 should be interpreted with caution. The stable performance observed in external validation (AUC = 0.90 for non‐AF and 0.80 for AF) further supports the robustness of the model at an inter‐institutional level within a single‐vendor environment, rather than cross‐platform generalizability. Previous studies have also emphasized classification approaches [12, 13, 14, 15, 16, 17, 18, 19, 20], and regression analyses, such as that by Vaid et al. [16], showed limited utility.

### 4.3 Comparison With DL‐Based AI Models

Despite recent progress, ECG‐based AI models using deep learning have not yet been widely adopted in clinical practice. Neural network–based DL approaches require large datasets and substantial computational resources, which are not always available in routine settings [12, 13, 14, 15, 16, 17, 18, 19, 20]. In addition, their “black‐box” nature limits interpretability and hinders clinical acceptance [18]. To address these issues, several groups have explored simplified approaches using structured ECG data. Hughes et al. reported that models based on 555 automated ECG measurements achieved performance comparable to DL‐based methods [26], and Katsushika et al. applied ML and DL techniques with 178 parameters to construct an explainable framework for predicting reduced LVEF [27]. While informative, these studies still depended on a large number of ECG features. In contrast, our study demonstrated that external validation was achievable using only age, sex, and 10 automatically derived ECG parameters. Using classical ML methods such as RF and XGBoost, the models achieved predictive performance comparable to prior AI‐ECG models while maintaining interpretability. Performance remained acceptable in patients with AF [18], and validation in two distinct hospital settings demonstrates inter‐institutional robustness, without implying universal generalizability. The identification of key predictors, including QRS duration, heart rate, QT interval, T‐axis, and RV5 voltage [11, 26], further indicates that classical ML can provide interpretable insights into ECG‐based LVEF prediction.

### 4.4 Clinical Implications

The simplicity of these predictive models has enabled the development of an application that can be used by a wide range of healthcare providers. By requiring only age, sex, and 10 automatically derived ECG parameters, the tool does not depend on manual measurements and can be applied consistently across institutions. It may be utilized not only by non‐cardiology specialists but also by co‐medical staff, and its usability extends to general clinics, routine health checkups, and other settings where advanced cardiovascular expertise is not readily available. The models implemented in the application (RF and XGBoost) were selected based on their superior performance in external validation, in line with the emphasis on clinical robustness. If an ECG can be recorded, this application may serve as a tool for initial screening. By reducing the workload of healthcare providers and minimizing unnecessary echocardiographic examinations, it has the potential to improve accessibility and efficiency in primary care, benefiting both patients and healthcare systems.

### 4.5 Study Limitations

This study has several limitations that must be acknowledged. First, although the dataset used for model development and validation was relatively large and diverse, it may not fully represent the variability observed in broader populations, particularly those from different geographic regions or healthcare systems. This limitation may have affected the generalizability of the findings beyond the study cohort. In addition, all ECGs used in this study were acquired exclusively from devices produced by a single manufacturer (Nihon Kohden, Japan), and the predictive models were constructed entirely based on automatically generated ECG parameters derived from this vendor's system. Because such automated measurements (e.g., QRS duration, QT/QTc, electrical axis, and voltage amplitudes) are known to vary across ECG manufacturers and even across different firmware versions, the transportability of our model to ECG data obtained from other vendors and platforms remains uncertain. Although we performed external validation using data from two independent institutions, this validation was conducted under a single‐vendor environment and should therefore be regarded as inter‐institutional reproducibility rather than true cross‐platform generalizability. Therefore, the present results should be interpreted with caution when applied to clinical settings using different ECG acquisition systems. Accordingly, further validation using ECG systems from different manufacturers will be required before broader clinical implementation can be considered. Second, the external validation cohort was obtained from a single institution. Although this ensured consistency in data quality, additional validation across multiple independent institutions will be necessary to enhance the applicability of predictive models across diverse settings. Third, the inherent variability in ECG acquisition, such as device‐specific differences and patient positioning, may have influenced the predictive performance of the models. This variability highlights the need for further standardization of data collection processes. Fourth, this study focused on identifying an LVEF of < 40% to optimize model performance and ensure comparability with prior AI‐ECG studies. Screening for mildly reduced EF (40–49%) is also clinically relevant and increasingly needed; however, the current model did not achieve sufficient performance when applying a 50% cutoff. Further development and validation of models capable of accurately detecting this intermediate range are important, and additional investigations will be required to achieve this goal. Finally, although user‐friendly application based on these models shows promise for clinical utility, its effectiveness in real‐world clinical settings has not yet been prospectively evaluated. Further studies are required to assess its impact on clinical decision–making and patient outcomes. In addition, because this was an observational study, causal inferences cannot be drawn. Moreover, our analyses were restricted to the prediction of reduced LVEF and did not directly examine downstream clinical outcomes.

## 5 Conclusion

We developed a simple machine learning model to estimate the likelihood of reduced LVEF (< 40%) using age, sex, and 10 automatically measured ECG parameters. The model demonstrated consistent performance in both internal and external evaluations, including patients with AF. This tool may provide a practical option for ECG‐based screening in clinical settings, although further validation across different ECG vendors and patient populations will be necessary before its broader generalizability can be fully established.

## Funding

The authors have nothing to report.

## Ethics Statement

The study was approved by the Research Ethics Committee of Ehime University Graduate School of Medicine (2312006).

## Conflicts of Interest

The authors declare no conflicts of interest.

## Supporting information


**Supplemental Figure 1** Scatter plots and regression lines for the relationship between individual ECG parameters and LVEF.
**Supplemental Figure 2**. Regression plots comparing predicted and observed LVEF values across internal (Ehime University Hospital) and external (Kitaishikai Hospital) validation cohorts for each machine learning model: GAMLASSO, SVM, RF, and XGBoost.
**Table S1:** Fold‐specific ROC AUC values from 10‐fold cross‐validation (internal validation cohort). mean (SD) = 0.91 (0.03); median (IQR) = 0.91 (0.90–0.92).
**Table S2:** Univariate ROC AUC values for each predictor (internal validation cohort)

## Data Availability

The data that support the findings of this study are available on request from the corresponding author. The data are not publicly available due to privacy or ethical restrictions.
