# Supplementary material for: Web‐Based Application of Simplified Machine Learning for Detecting Reduced LVEF From 12‐Lead ECG
Source: J Arrhythm. 2026 Feb 20;42(1):e70296. doi: 10.1002/joa3.70296 (PMC12928092; doi:10.1002/joa3.70296)
Supplement: Supplementary file 1 — Supplemental Figure 1 Scatter plots and regression lines for the relationship between individual ECG parameters and LVEF. Supplemental Figure 2. Regression plots comparing predicted and observed LVEF values across internal (Ehime University Hospital) and external (Kitaishikai Hospital) validation cohorts for each machine learning model: GAMLASSO, SVM, RF, and XGBoost. Table S1: Fold‐specific ROC AUC values from 10‐fold cross‐validation (internal validation cohort). mean (SD) = 0.91 (0.03); median (IQR) = 0.91 (0.90–0.92). Table S2: Univariate ROC AUC values for each predictor (internal validation cohort) [file JOA3-42-e70296-s001.docx]

**SUPPLEMENTAL MATERIAL**

**Web-Based Application of Simplified Machine Learning for Detecting Reduced LVEF from 12-Lead ECG**

**AUTHORS**

Hiroshi Kawakami, MD, PhD, Yohei Doi, MD, PhD, Kazumichi Yamamoto, MD, PhD, Yan Luo, MD, PhD, Makoto Saito, MD, PhD, MPH, Katsuji Inoue, MD, PhD, Osamu Yamaguchi, MD, PhD

**Abbreviations list**

AF, atrial fibrillation; AUC, area under the curve; DL, deep learning; ECG, electrocardiogram; LASSO, least absolute shrinkage and selection operator; LVEF, left ventricular ejection fraction; RMSE, root mean squared error; RF, random forest; SVM, support vector machine; TTE, transthoracic echocardiography; XGBoost, extreme gradient boosting.

**Supplemental Figure 1**. Scatter plots and regression lines for the relationship between individual ECG parameters and LVEF.


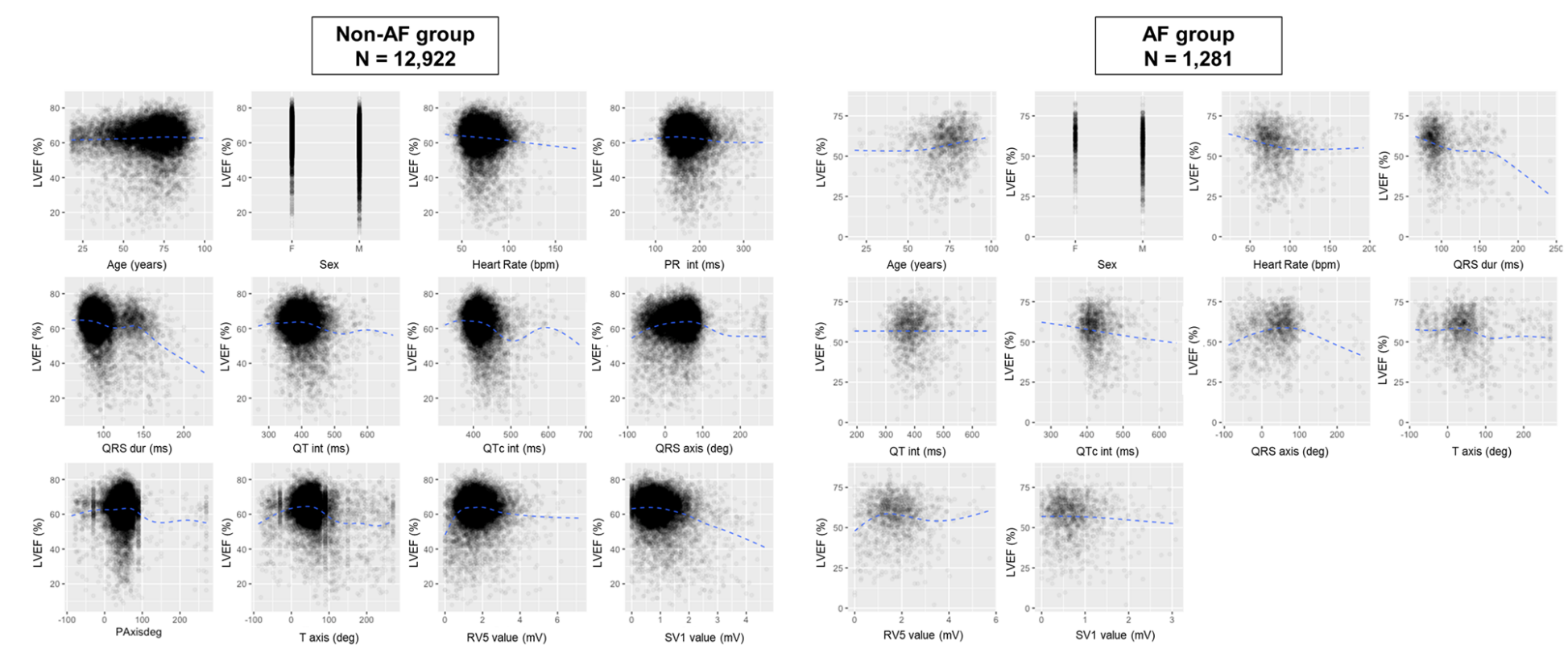


**Supplemental Figure 2**. Regression plots comparing predicted and observed LVEF values across internal (Ehime University Hospital) and external (Kitaishikai Hospital) validation cohorts for each machine learning model: GAMLASSO, SVM, RF, and XGBoost.


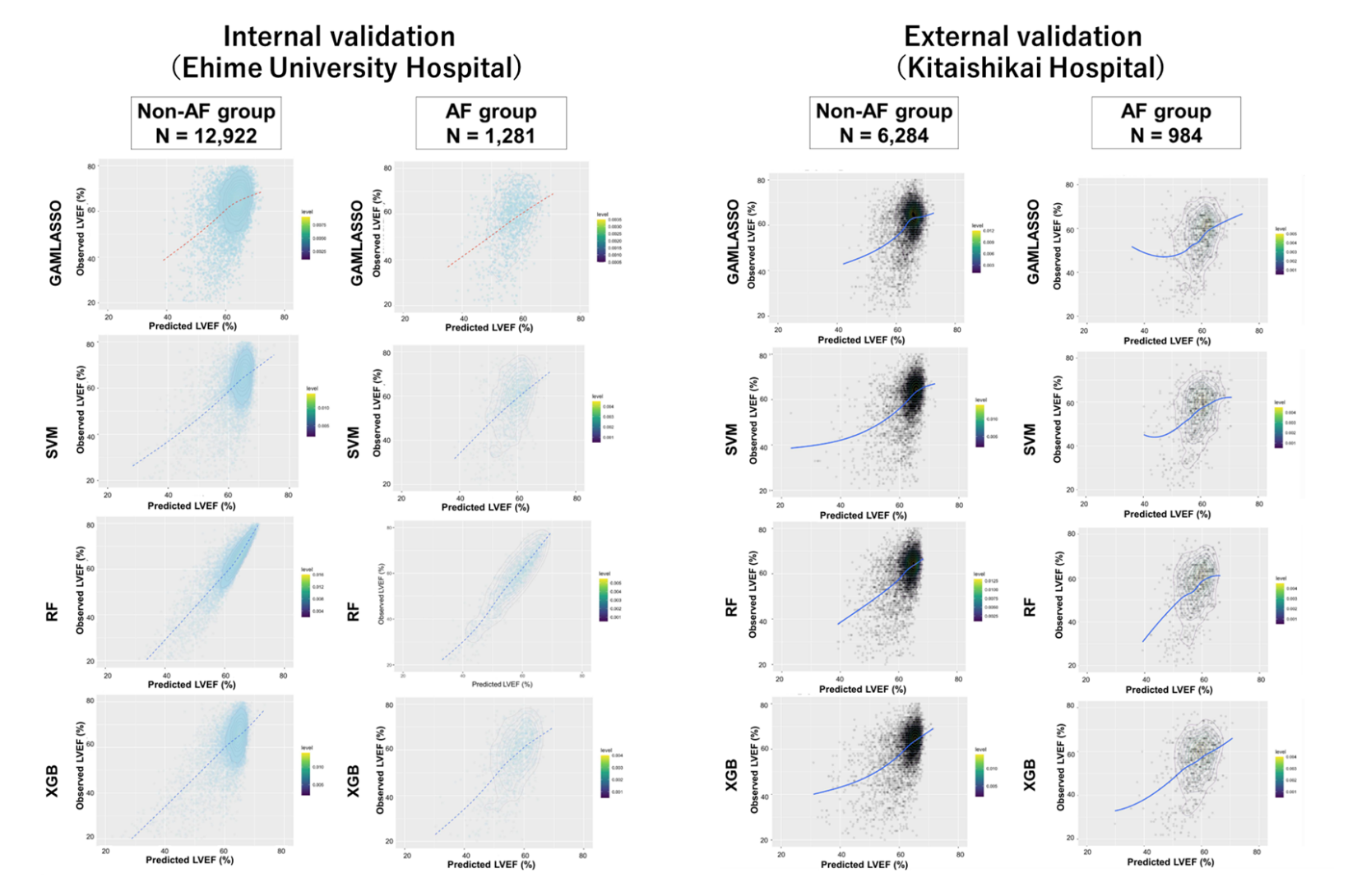


**Table S1. Fold-specific ROC AUC values from 10-fold cross-validation (internal validation cohort). mean (SD) = 0.91 (0.03); median (IQR) = 0.91 (0.90–0.92).**

| **Fold** | **ROC AUC** |
| --- | --- |
| Fold01 | 0.91 |
| Fold02 | 0.91 |
| Fold03 | 0.92 |
| Fold04 | 0.91 |
| Fold05 | 0.94 |
| Fold06 | 0.90 |
| Fold07 | 0.93 |
| Fold08 | 0.90 |
| Fold09 | 0.86 |
| Fold10 | 0.86 |

**Table S2. Univariate ROC AUC values for each predictor (internal validation cohort)**

| **Variable** | **ROC AUC** |
| --- | --- |
| Age | 0.53 |
| Sex | 0.38 |
| Heart rate | 0.59 |
| PR interval | 0.60 |
| QRS duration | 0.75 |
| QT interval | 0.65 |
| QTc interval | 0.77 |
| P axis | 0.52 |
| QRS axis | 0.61 |
| T axis | 0.72 |
| RV5 amplitude | 0.58 |
| SV1 amplitude | 0.67 |
